# Diverged subpopulations in tropical *Urochloa* (*Brachiaria*) forage species indicate a role for facultative apomixis and varying ploidy in their population structure and evolution

**DOI:** 10.1093/aob/mcac115

**Published:** 2022-09-16

**Authors:** J Higgins, P Tomaszewska, T K Pellny, V Castiblanco, J Arango, J Tohme, T Schwarzacher, R A Mitchell, J S Heslop-Harrison, J J De Vega

**Affiliations:** Earlham Institute, Norwich Research Park, Norwich NR4 7UZ, UK; Department of Genetics and Genome Biology, University of Leicester, Leicester LE1 7RH, UK; Department of Genetics and Cell Physiology, Faculty of Biological Sciences, University of Wroclaw, 50-328 Wroclaw, Poland; Rothamsted Research, Harpenden, Hertfordshire AL5 2JQ, UK; International Center for Tropical Agriculture (CIAT), 6713 Cali, Colombia; International Center for Tropical Agriculture (CIAT), 6713 Cali, Colombia; International Center for Tropical Agriculture (CIAT), 6713 Cali, Colombia; Department of Genetics and Genome Biology, University of Leicester, Leicester LE1 7RH, UK; Rothamsted Research, Harpenden, Hertfordshire AL5 2JQ, UK; Department of Genetics and Genome Biology, University of Leicester, Leicester LE1 7RH, UK; Key Laboratory of Plant Resources Conservation and Sustainable Utilization/Guangdong Provincial, Key Laboratory of Applied Botany, South China Botanical Garden, Chinese Academy of Sciences, Guangzhou, 510650, China; Earlham Institute, Norwich Research Park, Norwich NR4 7UZ, UK

**Keywords:** Grassland, breeding, forage, RNA-seq, apomixis, parthenogenesis, polyploidy, *Urochloa*, *Brachiaria*, Panicieae, *Megathyrsus*, *brizantha*, *humidicola*

## Abstract

**Background:**

*Urochloa* (syn. *Brachiaria*) is a genus of tropical grasses sown as forage feedstock, particularly in marginal soils. Here we aimed to clarify the genetic diversity and population structure in *Urochloa* species to understand better how population evolution relates to ploidy level and occurrence of apomictic reproduction.

**Methods:**

We explored the genetic diversity of 111 accessions from the five *Urochloa* species used to develop commercial cultivars. These accessions were conserved from wild materials collected at their centre of origin in Africa, and they tentatively represent the complete *Urochloa* gene pool used in breeding programmes. We used RNA-sequencing to generate 1.1 million single nucleotide polymorphism loci. We employed genetic admixture, principal component and phylogenetic analyses to define subpopulations.

**Results:**

We observed three highly differentiated subpopulations in *U. brizantha*, which were unrelated to ploidy: one intermixed with *U. decumbens*, and two diverged from the former and the other species in the complex. We also observed two subpopulations in *U. humidicola*, unrelated to ploidy; one subpopulation had fewer accessions but included the only characterized sexual accession in the species. Our results also supported a division of *U. decumbens* between diploids and polyploids, and no subpopulations within *U. ruziziensis* and *U. maxima*.

**Conclusions:**

Polyploid *U. decumbens* are more closely related to polyploid *U. brizantha* than to diploid *U. decumbens*, which supports the divergence of both polyploid groups from a common tetraploid ancestor and provides evidence for the hybridization barrier of ploidy. The three differentiated subpopulations of apomictic polyploid *U. brizantha* accessions constitute diverged ecotypes, which can probably be utilized in hybrid breeding. Subpopulations were not observed in non-apomictic *U. ruziziensis*. Sexual *Urochloa* polyploids were not found (*U. brizantha*, *U. decumbens*) or were limited to small subpopulations (*U. humidicola*). The subpopulation structure observed in the *Urochloa* sexual–apomictic multiploidy complexes supports geographical parthenogenesis, where the polyploid genotypes exploit the evolutionary advantage of apomixis, i.e. uniparental reproduction and clonality, to occupy extensive geographical areas.

## INTRODUCTION

*Urochloa* is a genus of tropical and subtropical grasses widely sown as forage to feed ruminants in the American and African tropics, particularly in areas with marginal soils. *Urochloa* grasses exhibit good resilience and low nutritional needs (Miles, 2007; Gracindo *et al.*, 2014; Maass *et al.*, 2015). The genus *Urochloa* includes species previously classified under *Brachiaria*, *Megathyrsus*, *Eriochloa* and *Panicum* (classification following Vorontsova and Simon, 2012; Torres González and Morton, 2005; Kellogg, 2015). Five species, *U. ruziziensis*, *U. decumbens*, *U. brizantha*, *U. humidicola* and *U. maxima* (guinea grass), are widely used as fodder plants, covering over 100 million hectares in Brazil alone (Jank *et al.*, 2014). Such an enormous area is half that of wheat or maize worldwide, which has a substantial environmental impact in terms of displacement of native species, water usage and provision of ecosystem services. In addition to extensive pasture systems in Latin America and Australia, *Urochloa* is planted in intensive smallholder systems in Africa and Asia (Keller-Grein *et al.*, 1996; Maass *et al.*, 2015). Breeding programmes in different countries have exploited the diversity among *Urochloa* spp. for the development of commercial forage cultivars by recurrent selection over many years (Jank *et al.*, 2014; Tsuruta *et al.*, 2015; Worthington and Miles, 2015).

Plant genetic resources provide the reservoir of adaptive and productive genes, usually free of deleterious mutations, to sustain crop genetic gain in breeding programmes. Joint missions between 1984 and 1985 conducted by the CGIAR (Consultative Group on International Agricultural Research) centres in several African countries collected native wild materials from the species in the genus, mostly as live plant cuttings or ramets (Keller-Grein *et al.*, 1996). These activities built a global grass collection with ~700 *Urochloa* accessions that are held at CIAT (Centro Internacional de Agricultura Tropical, Colombia), ILRI (International Livestock Research Institute, Kenya) and EMBRAPA (Brazilian Agricultural Research Corporation, Brazil).

Most *Urochloa* species are facultative apomicts, where asexual and sexual genotypes co-occur (Hörandl and Hojsgaard, 2012; Ortiz *et al.*, 2013; Hojsgaard and Hörandl, 2015). Residual sexuality, and a proportion of sexual Polygonum-type embryo sacs, can be expected among apomictic genotypes (Worthington *et al.*, 2016; Reutemann *et al.*, 2022). *Urochloa* diploids usually are sexual, but natural sexual polyploid *Urochloa* accessions are exceptionally uncommon. Reproduction mode (sexually or asexual) appears to be genetically determined in *Urochloa* genotypes (Worthington *et al.*, 2016). Apomixis is asexual (clonal) seed formation without chromosome reduction during meiosis and ploidy restitution by syngamy, resulting in progeny that is genetically identical to the parent. Highly successful apomictic genotypes can persist for hundreds of years, at the cost of having limited genetic variation and accumulating somatic mutations (Albertini *et al.*, 2019). Most apomictic angiosperm species are facultative (Albertini *et al.*, 2019), an evolutionary strategy that allows species to exploit the benefits of a quick and wide dispersion of successful clones during favourable conditions, while retaining the advantages of sexual segregation to adapt to changing habitats (Mangla *et al.*, 2015; Albertini *et al.*, 2019). Apomixis can result in divergent geographical distribution between sexual and asexual individuals, a scenario described as ‘geographical parthenogenesis’, in which apomicts colonize extensive geographical areas while sexual relatives are restricted to small refugees, followed by reversals to complete sexuality for the establishment of new populations (Hojsgaard and Hörandl, 2015). The consequences of apomixis and extent of genetic divergence among accessions can be accurately described through sequencing-based genotyping of populations from DNA or RNA samples, as here.

Apomictic or mixed apomictic and sexual reproduction in *Urochloa* spp. has resulted in varying ploidy levels and sub-genome structure within and among *Urochloa* species (Do Valle and Savidan, 1996; Keller-Grein *et al.*, 1996; Tomaszewska *et al.*, 2021*a*, *b*). In a recent study (Tomaszewska *et al.*, 2021*b*), we used flow cytometry to experimentally determine the ploidy of over 350 *Urochloa* accessions from the CIAT’s gene bank. Polyploidy is an important driver of plant evolution in natural populations and probably the most important mechanism of evolution of new species from an ancestor (sympatric speciation) in land plants (Otto and Whitton, 2000). Polyploidy can have benefits, such as heterosis, gene redundancy and loss of self-incompatibility, generating individuals that often cope better with fluctuating environments, exploit new niches or out-compete other species (Te Beest *et al.*, 2012).

Sub-genome variability and ploidy levels can be exploited for continued improvement through breeding. This variability has been successfully exploited in the improvement of other crop tribes, such as Triticeae and Brassicaceae (Gale and Miller, 1987; Burton *et al.*, 2004; Ali *et al.*, 2016). However, the genetic composition and relationships in *Urochloa* are poorly understood. Previous studies from countries with active *Urochloa* breeding programmes have explored the phylogeny in these species to inform breeding using ITS (internal transcribed spacer), RAPD (random amplified polymorphic DNA) and microsatellite markers (Torres González and Morton, 2005; Jungmann *et al.*, 2010; Vigna *et al.*, 2011*a*, *b*; Ferreira *et al.*, 2016; Triviño *et al.*, 2017). However, previous studies disagree on the number of subpopulations in *U. brizantha* (Vigna *et al.*, 2011*b*; Triviño *et al.*, 2017) and *U. humidicola* (Vigna *et al.*, 2011*a*; Triviño *et al.*, 2017; Worthington *et al.*, 2019), the relationship of *U. decumbens* to *U. ruziziensis* (Ambiel *et al.*, 2008; Ferreira *et al.*, 2016; Triviño *et al.*, 2017), and the inclusion or not of guinea grass in the genus *Urochloa* (Triviño *et al.*, 2017; Tomaszewska *et al.*, 2021*b*).

Three *Urochloa* species (*U. brizantha*, *U. decumbens* and *U. ruziziensis*) have been assigned to an agamic ‘brizantha complex’ (Do Valle and Savidan, 1996; Renvoize *et al.*, 1996; Ferreira *et al.*, 2016; Triviño *et al.*, 2017). Crosses between ten founders, eight *U. brizantha*, one *U. decumbens* (cv. Basilisk) and one synthetic autotetraploid *U. ruziziensis* (BRX 44-02) were completed in the late 1980s, and their progeny constitutes the gene pool of the recurrent selection breeding programme at CIAT targeting this species complex (Miles *et al.*, 2006). A similar breeding scheme is used at EMBRAPA (Barrios *et al.*, 2013), but we could not find information on the founders. On the other hand, *U. humidicola* and *U. dictyoneura* have been arranged in the ‘humidicola complex’ (Lutts *et al.*, 1991; Renvoize *et al.*, 1996; Triviño *et al.*, 2017). More recently, independent hexaploidy *U. humidicola* breeding programmes have also been established at CIAT and EMBRAPA after the discovery in the mid-2000s of a natural sexual polyploid germplasm accession that could be crossed with apomictic polyploid *U. humidicola* pollen donors (Jungmann *et al.*, 2010; Vigna *et al.*, 2011*a*). Finally, guinea grass is also known as *Megathyrsus maximus*, *Panicum maximum* or *Urochloa maxima.* Two over-performing wild accessions of *U. maxima*, namely cv. Tanzania and cv. Mombaça, collected in East Africa in the 1970s are responsible for 10 % of the total forage seed market in Brazil, but there is also an active breeding programme at EMBRAPA (Jank *et al.*, 2014). Recently, 90 candidate parental males were selected by phenotypic analysis for test crosses for a breeding programme on *U. maxima* starting at CIAT (Villegas *et al.*, 2020).

We used a diversity panel of 111 accessions, which are representative of the collections of wild materials in Africa in 1984 and 1985. These 111 accessions belong to the five *Urochloa* spp. that are used in the development of commercial forage cultivars. We used RNA-sequencing (RNA-seq) from total RNA, so tentatively representing the genetic diversity within the complete *Urochloa* gene pool used in breeding worldwide. This work is supported by the genome assembly and gene annotation of the diploid accession 26162 (2*n* = 2*x* = 18) of *U. ruziziensis* (GCA_003016355) that we recently made available (Worthington *et al.*, 2021). It has allowed greater use of genomic approaches to characterize these materials. For example, we identified loss-of-function (LOF) genes related to forage quality and environmental impact using allele mining (Hanley *et al.*, 2021).

The objectives of our study were to obtain a comprehensive picture of (1) the available diversity in *Urochloa* species, (2) the population structure and evolution (ancestors and divergence) in the different species complexes, and (3) how population structure and evolution relate to ploidy level and reproduction mode of the genotypes within each group. The impact of this knowledge will lead to (1) a better understanding of the genetic diversity of *Urochloa* genetic resources held in genebanks, (2) the ability to exploit ploidy levels and subgenome composition in breeding (following the example of other crop tribes), and (3) the potential to establish heterotic groups and ecotypes which would benefit from hybrid vigour and give rise to novel adaptive traits in recombinant populations.

## MATERIALS AND METHODS

### RNA extraction and sequencing

We sequenced 111 accessions from five *Urochloa* (including some species previously included in *Brachiaria*, *Panicum* and *Megathyrus*) species. Leaf material from 104 accessions was sampled on the same day from the *ex situ* field collection maintained by the Genebank at CIAT in Cali, Colombia. Accessions sourced from CIAT are named as, for example, ‘CIAT 26146’, but we have removed ‘CIAT’ from our text. Fresh leaf material was collected and immediately frozen in liquid nitrogen. Tissue samples were ground in liquid nitrogen and lyophilized. Total RNA was extracted as described in Hanley *et al.* (2021). Another seven accessions were obtained from the United States Department of Agriculture (USDA, GA, USA) as seeds. These seven accessions include ‘PI’ at the beginning of their ID. These seven accessions were sampled at a different time than the other accessions after growing in glasshouses at the University of Leicester, UK. We generated a single sample from each accession, and we use ‘sample’ and ‘accessions’ as synonyms throughout the text. For all tissue samples, Illumina sequencing using standard RNA-seq library preparations with 150-bp paired reads was conducted by Novogene Europe (Cambridge, UK). The raw reads were deposited in SRA under Bioproject PRJNA513453.

### Read alignment and SNP calling

Raw reads were pre-processed using Trim galore v.0.5 (Krueger *et al.*, 2021) with the options for Illumina paired reads and trimming 13 bp at the 5ʹ end in both reads. Processed reads were aligned to the available *Urochloa* genome (Worthington *et al.*, 2021), which corresponded to the *U. ruziziensis* accession 26162 (2*n* = 2*x* = 18). RNA to DNA alignments were done using STAR v.2.6.0c (Dobin *et al.*, 2013) with a minimum overlap of 30 % and a maximum mismatch of 3 bp per alignment, in order to allow for mapping from more distant species to the genome. Alignment coverage was calculated using BEDTools genomecov. Single nucleotide polymorphism (SNP) calling was done using GATK v.3.7.0 and the recommended pipeline for RNA-seq (Van der Auwera *et al.*, 2013). First, we used PicardTools v.2.1.1 to annotate duplicate reads using the option MarkDuplicates. Later, we used GATK’s tool SplitNCigarReads with the options ‘-rf ReassignOneMappingQuality -RMQF 255 -RMQT 60 -U ALLOW_N_CIGAR_READS’ to reformat some alignments that span introns to match conventions for the final step. The final step was SNP calling using GATK’s tool HaplotypeCaller with all the sequenced accessions (samples) at the same time (multisample mode). SNP calling was run with the options ‘-ploidy 6 -dontUseSoftClippedBases -stand_call_conf 20 -maxNumHaplotypesInPopulation 128’ to obtain a good quality calling from RNA alignments. After GATK, SNPs were filtered for quality, clustering (close SNP loci) and minor allele frequency (MAF) of 1 %. Then, calls with a depth <5 were set to missing and immediately sites with >40 % missing data were removed to obtain the final set. Two additional subsets were obtained by filtering out the 67 accessions in the agamic group and the *U. humidicola* accessions. These subsets were filtered for an MAF of 1 %.

### Population analysis

Population structure analysis was performed through ADMIXTURE (Alexander and Lange, 2011) using *K *= 3 to *K *= 10 for the 111 accessions, *K *= 2 to *K *= 8 for the 67 samples in the agamic group (*U. ruziziensis*, *U. decumbens* and *U. brizantha*) and *K *= 2 to *K *= 8 for the 28 *U. humidicola* accessions. Each value of *K* was run ten times, and the cross-validation error was averaged over the ten runs. The ten output files were combined using CLUMPP within the R package POPHELPER v.2.2.7 (Francis, 2017). Principal component analysis (PCA) was carried out using Tassel v.5.2.41 (Bradbury *et al.*, 2007). A UPGMA (unweighted pair group method with arithmetic mean) hierarchical phylogenetic tree was built with Tassel v.5.2.41 (Bradbury *et al.*, 2007) and plotted with iTOL v.6.5.2 (Letunic and Bork, 2007).

## RESULTS

### *Sequencing, aligning and SNP calling in a panel of* Urochloa *accessions from five species*

**Fig. 1.**
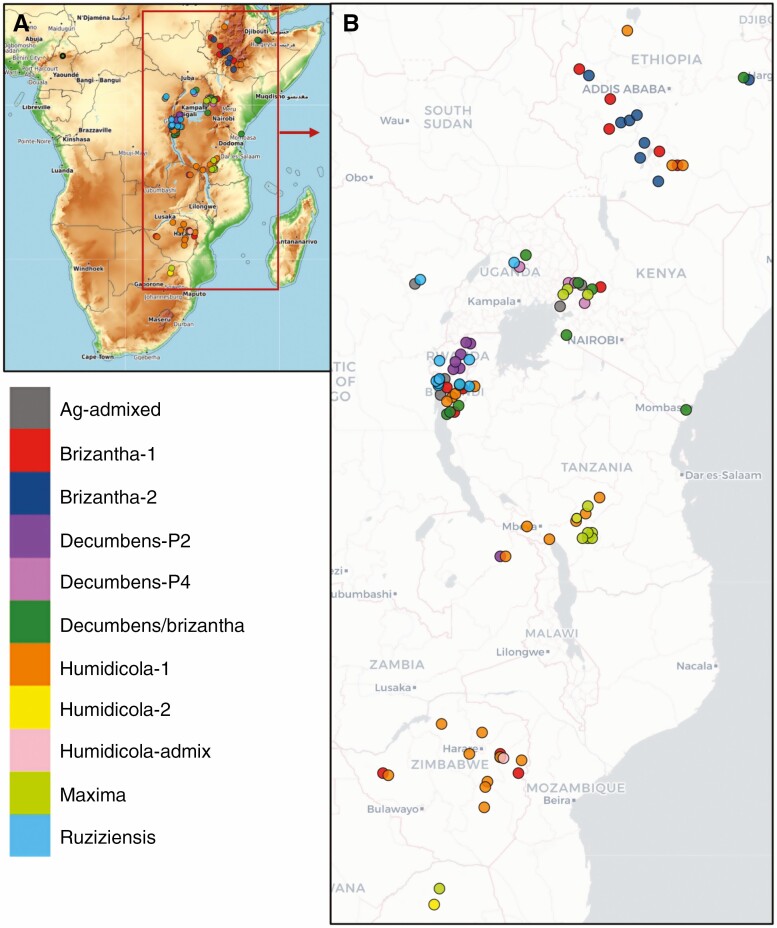
Geographical origin of 92 *Urochloa* accessions with collection coordinates (74 accessions) or country of origin (18 accessions). Accessions are coloured by subpopulation. (A) Origin in sub-Sahara Africa. (B) Greater detail for East Africa. (C) Greater detail for the Great Lakes region.

We sequenced 111 accessions from five *Urochloa* (syn. *Brachiaria*) species: *U. ruziziensis*, *U. brizantha*, *U. decumbens*, *U. humidicola* and *U. maxima* (syn. *Megathyrsus maximus*). Species identity and ploidy were previously determined using plant architecture traits and flow cytometry of fluorescently stained nuclei (Tomaszewska *et al.*, 2021*a*, *b*). The country of origin of 92 accessions was known, and for 75 accessions we also knew the collection coordinates (Fig. 1). Accessions were collected in a broad range of latitudes (20.08°S to 11.37°N) but not of longitudes (26.98°E to 42.05°E), except for one *U. brizantha* accession from Cameroon. Annotations are summarized in Table 1 (and detailed in Supplementary Data Table S1).

**Table 1.** Summary of the species (rows) and ploidy level (columns) of the 111 accessions used in this study. Details of each accession are given in Supplementary Data Table S1.

	2*n*	4*n*	5*n*	6*n*	7*n*	8*n*/9*n*	Unknown	Total
*U. ruziziensis*	10						1	11
*U. decumbens*	8	17					1	26
*U. brizantha*		17	9	2			1	29
*U. humidicola*				10	16	2		28
*U. maxima*		12					1^*^	13
*U.* hybrid		3	1					4^†^

Sample 28 was labelled as a *U. humidicola* accession, but it corresponds to an unknown *U. maxima* accession instead, based on our results.

Accession BR02/1752 (cv. Cayman) clustered within the agamic group.

**Fig. 2.**
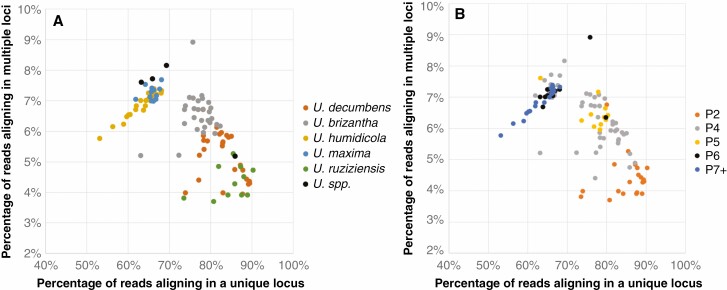
Percentage of reads aligning in either uniquely or in multiple positions in the *Urochloa* reference genome from Worthington *et al.* (2021). The 111 accessions are coloured by species (A) or ploidy (B).

RNA-seq reads from the accessions were aligned to the available *Urochloa* genome assembly and gene annotation (Worthington *et al.*, 2021), which corresponds to the diploid *U. ruziziensis* accession 26162 (2*n* = 2*x* = 18). Two well-defined groups of species were observed based on aligning metrics (Fig. 2): (1) accessions where >70 % of the reads aligned in one unique locus to the reference genomes, namely *U. ruziziensis* (71–87.2 %), *U. decumbens* (70.8–86.5 %) and *U. brizantha* (69.7–79.4 %; excluding accession 16152 that had a value of 60.9 %); and (2) accessions where <70 % of the reads aligned in one unique locus to the reference genomes, namely *U. maxima* (60–66.1 %) and *U. humidicola* (51.4–65.9 %) (Fig. 2A). The grouping was correlated to the genetic distance to the reference genome (reference bias).

The percentage of reads mapping in multiple loci increased with ploidy (Fig. 2B) for the group of the accessions belonging to the species *U. ruziziensis*, *U. decumbens* and *U. brizantha*; diploids had a percentage of reads mapping in multiple loci <5 %, while it was >5 % in most polyploid accessions. However, the percentage of reads mapping in multiple loci in the other species, which are more distant to the reference species, was directly proportional to the total number of mapped reads (Fig. 2B), i.e. not correlated with ploidy.

RNA-seq reads covered 268.84 Mb (~36.7 % of the 732.5 Mb genome assembly). The covered regions are more than 2.5 times the original gene annotation from the *U. ruziziensis* genome (43 152 genes comprising 102 Mb). The median read coverage was 25 reads in the covered regions, and the average read coverage in these regions was 2587 ± 54 293 reads. This is also observed in differential gene expression experiments because a few genes are very highly expressed. GATK identified 6 461 493 variants, which included 5 757 116 SNPs. These were filtered to give a final set of 1 167 542 SNPs. Two additional subsets were obtained by filtering out either the 67 accessions in the agamic group (895 667 SNPs) or the *U. humidicola* accessions (512 611 SNPs). After SNP calling and filtering, the average SNP density in the genome was 7.3 SNPs/kb. Using the 43 152 genes and 202 681 exons annotated in the genome reference, the median was 69 and 13 SNPs per gene and exon, respectively (average was 95 and 36 per gene and exon, respectively). In total, 34 981 of the annotated genes had at least one SNP.

### Admixture analysis

**Fig. 3.**
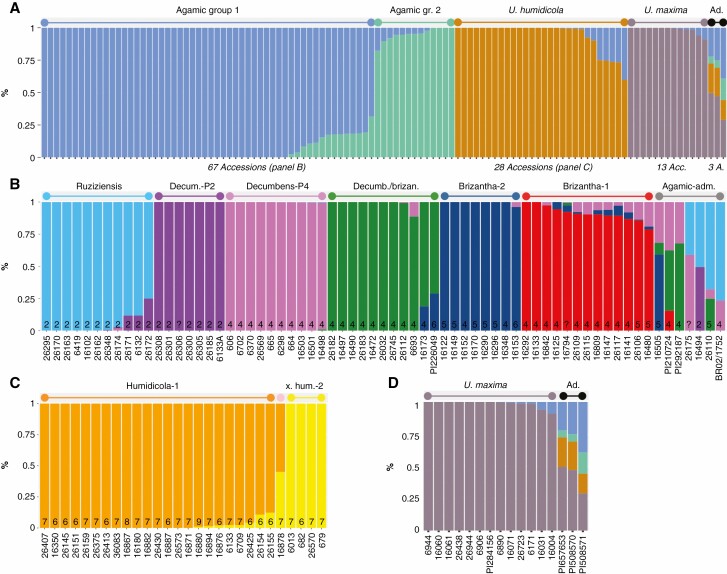
Admixture analysis of the genetic ancestry inferred in the complete set of 111 *Urochloa* accessions (A), the subset of 67 accessions in the agamic group (B) and the subset of 28 *U. humidicola* accessions (C). Ploidy level is given at the foot of each column. Each accession is represented by a stacked column partitioned by the proportion of the ancestral genetic component, where each identified ancestral genetic component is represented by a different colour. Accessions with a single colour are ‘pure’. A minimum threshold of 50 % (A) or 70 % (B and C) genetic composition was used to assign accessions to groups. In A: ‘gr.’ means group, while ‘acc.’ and ‘a.’ mean accession. In B: ‘adm.’ means admixed. In C, ‘x’ means ‘humidicola-admix’, and ‘hum.-2’ means ‘humidicola-2’.

**Fig. 4.**
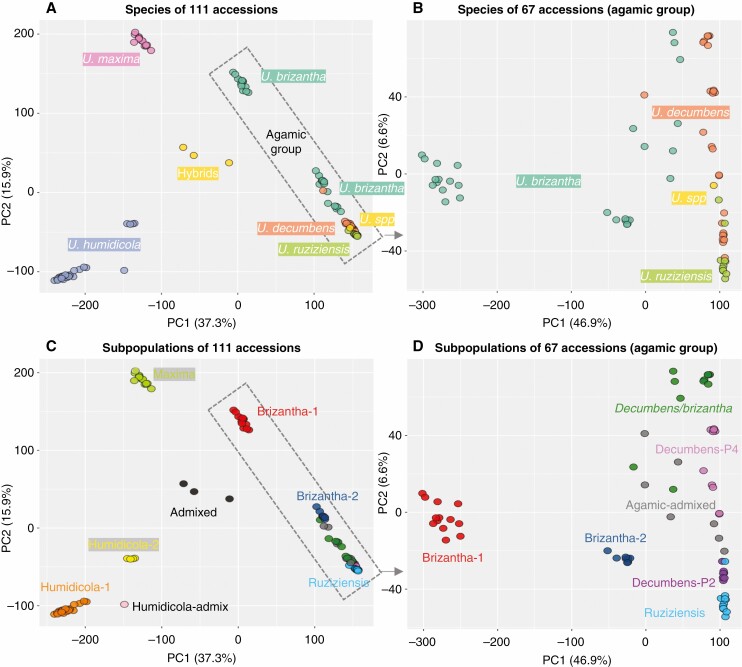
Population structure by principal component analysis (PCA) using the top two components to separate the complete set of 111 accessions (A and C) or to separate the subset of 67 accessions in the agamic group (B and D). Accessions are coloured by species (A and B) or subpopulation (C and D). *U. spp* = *Urochloa* hybrid cv. Cayman (BR02/1752).

We used genetic admixture analysis to define subpopulations (Fig. 3). The ‘admixture model’ assumes that each individual has ancestry from one or more of ‘*K*’ genetically distinct sources. An estimation of four subpopulations (*K* = 4) was selected based on the CV error (Supplementary Data Fig. S1A) and population structure. To assign 111 *Urochloa* accessions to the subpopulations identified, the admixture (Fig. 3) and principal component (Fig. 4) analyses were considered together. A minimum threshold of 50 % genetic composition was used to assign accessions to groups. This allowed us to place the accessions in four groups (Fig. 3): *U. humidicola* (28 accessions), *U. maxima* (13 accessions), ‘agamic group 1’ (54 accessions from the three remaining species) and a closely related ‘agamic group 2’ (that corresponded to the ‘brizantha-1’ subpopulation). Three accessions obtained from USDA and identified simply as ‘*Urochloa* sp.’ showed an admixture of these four groups and were annotated as ‘admixed’ (Fig. 3). Sample 86 was received as *U. humidicola* (Accession 26438). However, it corresponds to an unknown accession that clearly clustered with the *U. maxima* accessions. Since 26438 has been verified as *U. humidicola* in previous studies (Triviño *et al.*, 2017), this is probably a mislabelling. When we reduced the number of groups (*K* = 3), the *U. humidicola* and *U. maxima* species clustered together, but the agamic groups ‘1’ and ‘2’ were consistent (Fig. S2). When we increased the number of groups (*K* = 5), a new group split from the ‘agamic group 1’ (that corresponded to the ‘brizantha-1’ subpopulation). The 28 accessions in the *U. humidicola* group had a basic chromosome number of 6 and high ploidy levels ranging from 6 to 9. The 12 accessions in the *U. maxima* group had a basic chromosome number of 8 and are tetraploid. The 67 accessions in the agamic groups had a basic chromosome number of 9 and ploidy levels ranging from 2 to 6 (Tomaszewska *et al.*, 2021*b*).

**Fig. 5.**
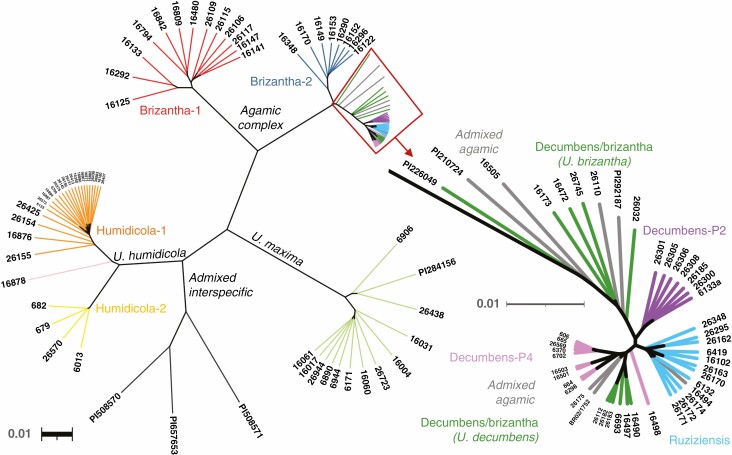
Phylogenetic tree using UPGMA hierarchical clustering of the complete set of 111 accessions separated the accessions into the two species complexes plus *U. maxima*, and into subpopulations following a similar division to the PCA and admixture analysis, except with *U. maxima* divided into two branches, and the ‘decumbens/brizantha’ subpopulation split by species. The tree on the right is a zoom in the branches included in the red box.

The admixture analysis was subsequently carried out using only the 67 accessions in the agamic group (Fig. 3B). An estimation of six groups (*K* = 6) was selected based on the CV error (Supplementary Data Fig. S1B) and population structure (Fig. 4). A minimum threshold of 70 % shared genetic composition was used to assign accessions to each of the six groups. The group ‘ruziziensis’ was composed of all 11 *U. ruziziensis* accessions (Fig. 3B). It included accession 6132 (sample 31), which was wrongly classified as *U. decumbens* (Fig. 5). Within it, five accessions showed shared ancestry (1–25 %) with diploid *U. decumbens*. All seven diploid *U. decumbens* accessions composed the group ‘decumbens-P2’ and were pure accessions with no shared ancestry with any other group. Similarly, ten tetraploid *U. decumbens* formed the group ‘decumbens-P4’ with pure accessions with no shared ancestry with any other group. However, another six tetraploid *U. decumbens* composed a different group together with five *U. brizantha* accessions, which was called ‘decumbens/brizantha’. This group of 11 accessions was the only one composed of more than one species. Despite this mix, these accessions showed clear shared ancestry among them and no shared ancestry with any other group (except two accessions with minor components). Finally, the groups ‘brizantha-1’ and ‘brizantha-2’ were formed by eight and 13 *U. brizantha* accessions, respectively. The group ‘brizantha-2’ has pure accessions with no shared ancestry with other groups (with one minor exception under 5 %), while most accessions in ‘brizantha-1’ have shared ancestry with ‘decumbens-P4’. The group ‘brizantha-1’ corresponds to the previous ‘agamic group 2’. The ‘brizantha-2’ subpopulation was only observed in Ethiopia, while ‘brizantha-1’ was observed in a broad range of latitudes. When we reduced the number of groups (*K* = 5), the ‘brizantha-decumbens’ merged with the ‘decumbens-P4’. When we increased the number of groups (*K* = 7), five ‘brizantha-1’ split into a subpopulation different in nature (Fig. S3).

The admixture analysis was finally completed using only the 28 *U. humidicola* accessions (Fig. 3C). An estimation of two groups (*K* = 2) was selected based on the CV error (Supplementary Data Fig. S1C) and population structure (Fig. 4). A minimum threshold of 70 % shared genetic composition was used to assign accessions to a group. The 28 accessions were assigned to two groups: 23 accessions into ‘humidicola-1’ and four accessions into ‘humidicola-2’. Accession 16878 was an equal mix from both *U. humidicola* groups and annotated as ‘humidicola-admixed’, i.e. a natural hybrid between both subpopulations. When we increased the number of groups (*K* = 3 and K = 4), we obtained a small subpopulation with the accessions with higher admixture (16878 and 26155) and an artificial split with some ‘humidicola-1’ accessions in an additional group (Fig. S4).

A smaller number of 13 *U. maxima* accessions showed little genetic diversity compared to the other species. At *K* = 4 (Fig. 3), these accessions formed one of the clearly defined subpopulations, but had a similar genetic make-up to each other. Because of the low diversity, we assigned all the *U. maxima* to a single subpopulation, named ‘maxima’.

Our analysis supports that the ‘agamic-admixed’ are (1) *U. decumbens* × *U. brizantha* (16505, PI210724 and PI292187, which were wrongly annotated in their passport data as *U. brizantha*, *U. decumbens* and *U. brizantha,* respectively); (2) *U. ruziziensis* × *U. decumbens* (26175 and 16494, which were respectively annotated as *U. ruziziensis* and *U. decumbens*; and BR02/1752 cv. Cayman); or (3) *U. ruziziensis *× *U. brizantha* (26110, wrongly annotated as *U. brizantha*).

### Population structure by PCA

A PCA showed the relationship between the 111 accessions, species and admixture groups (Fig. 4A, C). The PCA was also done for the 67 accessions in the agamic group alone (Fig. 4B, D). PCA allowed us to define 12 clusters in total, which closely corresponded to the ten subpopulations and two admixed groups. The distribution of accessions into subpopulations according to the species and ploidy annotations is represented in Supplementary Data Fig. S5.

All subpopulations contained accessions from a single species, except subpopulation ‘decumbens/brizantha’. Notably, this subpopulation contained accessions that showed greater similarity to each other – regardless of species – than to accessions from the same species in different subpopulations. The two diploid subpopulations, ‘decumbens-P2’ and ‘ruziziensis’ clustered together and apart from polyploid subpopulations. Subpopulation ‘brizantha-1’ was distant from other agamic subpopulations, including ‘brizantha-2’. However, accessions in ‘brizantha-1’ showed shared admixture with tetraploid *U. decumbens*, while accessions in ‘brizantha-2’ did not. Numerically, PC1 in Fig. 4B, which separates ‘brizantha-1’ from the rest of the agamic complex, explains 46 % of the diversity. PC2, which separates the subpopulations within the agamic complex, explains 6.6 %. There is very low variation in the agamic group once brizantha-1 is excluded.

Two groups of accessions contained hybrids, one with the hybrids between the distant *Urochloa* species (‘admixed’ subpopulation) and the other with the hybrids within the three species in the agamic group (‘agamic-admixed’).

### Population structure by phylogenetic analysis

We built a phylogenetic tree using UPGMA hierarchical clustering and plotted it un-rooted (Fig. 5). The clustering matches the PCA and admixture analysis in general terms, with the following differences: (1) *U. maxima* divided into two branches, with three of the *U. maxima* accessions (6906, the unknown accession we received as 26438 and PI284156) separated from the rest; (2) the ‘decumbens/brizantha’ subpopulation split by species, with *U. decumbens* within the subpopulation clustering close to the other polyploid *U. decumbens* but *U. brizantha* within the subpopulation placed in between brizantha-2 and the other subpopulations in the agamic complex – the latter was also observed in the PCA; and (3) accessions admixed from the three species in the agamic complex (‘agamic-admixed’) spread among the subpopulations.

## DISCUSSION

We defined the population structure and evolution between and within five *Urochloa* spp. that are used in the development of commercial forage cultivars. By using RNA-seq, we utilized an unprecedented number of markers, over 1.1 million SNP loci, that covered most of the complete transcriptome from the accessions based on the total genome length covered by the reads (~269 Mb or 37 % of the genome). We obtained a median of 69 and 13 SNP sites per gene and exon, respectively, which makes this dataset a valuable resource for breeders and researchers.

### *A single polyploidization event probably established polyploid* U. brizantha *and* U. decumbens

The two *U. decumbens* subpopulations were divided by ploidy. Diploid *U. decumbens* are closely related to *U. ruziziensis*, while polyploid *U. decumbens* are closely related to *U. brizantha.* This split in two *U. decumbens* subpopulations by ploidy was previously reported using microsatellites (Triviño *et al.*, 2017). In previous studies, the relationship of *U. decumbens* with the other two species has been discussed, as it was alternatively found to be closely related to *U. ruziziensis* (Ferreira *et al.*, 2016) or *U. brizantha* (Ambiel *et al.*, 2008). In fact, both observations were correct depending on the ploidy of the accessions under consideration. Finding that polyploid *U. decumbens* are more closely related to *U. brizantha* than to diploid *U. decumbens* adds support to an evolutionary model (previously proposed) where polyploidization established a tetraploid ancestor, from which *U. brizantha* and polyploid *U. decumbens* diverged.

### *Three* U. brizantha *subpopulations probably constitute diverged ecotypes*

*U. brizantha* diversity is complex and probably divided into several ecotypes. A group of 11 *U. brizantha* accessions was different to the rest of the agamic group to form a readily distinguishable cluster (‘brizantha-1’ or ‘agamic group 2’). This group is clearly different, as visualized in the phylogenetic tree (Fig. 5) or evidenced numerically by PC1 in the PCA, which separates ‘brizantha-1’ from the other subpopulations of the agamic group and explains 47 % of the variation among these species (Fig. 4B). Despite ‘brizantha-1’ being distant from the other species in the agamic complex, we observed admixture between ‘brizantha-1’ and *U. decumbens* and other *U. brizantha* (Fig. 3B).

We also observed a subpopulation, named ‘decumbens/brizantha’, that included an almost equal number of *U. decumbens* and *U. brizantha* accessions. This is the only subpopulation with more than one species in our study. Multiple evidence supported this is a distinct subpopulation: (1) admixture analysis (Fig. 3B) showed shared ancestry within the group and different to any other subpopulation (Fig. 3B); and (2) PCA and phylogenetic analysis showed ‘decumbens/brizantha’ clustered apart from other groups (Figs 4D and 5). The ‘decumbens/brizantha’ subpopulation held greater genetic diversity than the other subpopulations as would be expected due to its interspecific nature. The *U. decumbens* within the subpopulation clustered close to ‘decumbens-P4’ in the phylogenetic analysis (Fig. 5) and merged with ‘decumbens-P4’ in an admixture analysis with fewer subpopulations (*K* = 5). At the same time, two *U. brizantha* accessions within the subpopulation (16173 and PI226049) shared ancestry with ‘brizantha-2’ and were situated ‘halfway’ between the subpopulations ‘decumbens/brizantha’ and ‘brizantha-2’ in the PCA (Fig. 4D) and phylogenetic analysis (Fig. 5).

Two findings, the shared ancestry but strong genetic differentiation of ‘brizantha-1’ and the interspecific *U. decumbens*/*U. brizantha* subpopulation, support an evolutionary scenario where a single polyploidization event established both the tetraploid *U. brizantha* and *U. decumbens*, as previously proposed by Pessoa-Filho *et al.* (2017) and Tomaszewska *et al.* (2021*b*) based on chromosomal and repetitive DNA analysis. This would be followed by the divergence of ‘brizantha-1’ by evolutionary processes putatively driven by adaptation and its facultative apomictic nature. The ‘brizantha-1’ subpopulation was observed over a broad range of latitudes (e.g. in Ethiopia and Zimbabwe), while ‘brizantha-2’ was only observed in Ethiopia. Apomixis (asexual reproduction) can result in divergent geographical distribution between sexual and asexual individuals, a scenario described as ‘geographical parthenogenesis’, in which apomicts colonize extensive geographical areas while sexual relatives are restricted to small refugees, followed by reversals to complete sexuality for the establishment of new populations (Hojsgaard and Hörandl, 2015).

Our results harmonized previous contradictory results. Vigna *et al.* (2011*b*) divided *U. brizantha* into three clusters after evaluating 172 accessions from EMBRAPA’s collection (sourced from the same fieldwork in the 1980s as the germplasm in the present study) using 20 SSR (simple sequence repeat) markers. However, these three clusters did not correspond to ours. Based on 11 accessions common between both studies, we inferred that our subpopulations ‘brizantha-1’ and ‘brizantha-2’ matched with their clusters II and I, respectively, but their cluster III included additional ‘brizantha-1’ and ‘brizantha-2’ accessions (e.g. 16122, 16480). Triviño *et al.* (2017) divided *U. brizantha* into two groups using UPGMA clustering based on 39 microsatellites: one group with fewer individuals and clustering close to the *U. decumbens* and admixed accessions that would correspond to ‘decumbens/brizantha’; and a larger group that included all the remaining *U. brizantha*. Most of the accessions we sequenced were included in Triviño *et al.* (2017). While they did not discuss a further division in *U. brizantha*, we observed that all our ‘brizantha-1’ accession clustered together in the left branches of the phylogenetic tree and all ‘brizantha-2’ clustered on the right branches of the tree.

### *Sexual reproduction was only found in the smallest of the two* U. humidicola *subpopulations, and is limiting in all* Urochloa spp.

We observed two different subpopulations in *U. humidicola*, ‘humidicola-1’ and ‘humidicola-2’, plus a single accession (16878) that was an equal mix from both subpopulations.

Combining our results with some of the results from Triviño *et al.* (2017) and Vigna *et al.* (2011*a*) adds support to the division of *U. humidicola* into two subpopulations, where ‘humidicola-2’ is significantly less common than ‘humidicola-1’, at an approximate ratio of 5 : 1. It also supports that the only known sexual accession (26146) is a ‘humidicola-2’ accession. In detail, Triviño *et al.* (2017) previously observed two subpopulations: a large group of *U. humidicola* accessions including all except three accessions. These three separate *U. humidicola* accessions were 675, 679 and 26146. Accession 679 is a ‘humidicola-2’ subpopulation in our study, and accession 26146 is the sexual *U. humidicola* accession that allowed the establishment of breeding programmes in the mid-2000s. Vigna *et al.* (2011*a*) analysed 26 *U. humidicola* accessions and used UPGMA clustering based on 38 microsatellites to divide *U. humidicola*. All seven common accessions in our study were ‘humidicola-1’ and appeared in the top branch of the tree. The bottom branch corresponds to ‘humidicola-2’, since it included the sexual accession 26146, one accession (26149) not sequenced in our dataset and the progeny from their crossing.

Taken together, the known ‘humidicola-2’ accessions are 26146 (Burundi), 26149 (Burundi), 6013 (South Africa), and 26570, 675, 679 and 682 (all from unknown origin). Since natural sexual accessions are scarce and limited to this significantly smaller subpopulation, we hypothesize that ‘geographical parthenogenesis’, where apomicts colonize extensive geographical areas while sexual relatives are restricted to small refugees, is probably the main driver of population divergence in *U. humidicola*.

The scarcity of sexual genotypes is not exclusive to *U. humidicola* but probably similar in the other *Urochloa* species, where natural sexual polyploid *Urochloa* accessions are exceptionally uncommon. Focusing on the species in our study: all *U. ruziziensis* are sexual and diploid; there is only one sexual *U. brizantha* accession, the diploid BRA 002747 (Rodrigues *et al.*, 2003; Silveira *et al.*, 2009), but there are several experimentally verified sexual diploid *U. decumbens* (e.g. 26308 and 26301). Because there are no known natural sexual polyploids in the *U. ruziziensis*, *U. brizantha*, and *U. decumbens* agamic complex, synthetic autotetraploid sexual genotypes were obtained with colchicine treatment of the diploid accessions to support breeding (Swenne *et al.*, 1981; Pinheiro *et al.*, 2000; Simioni and Valle, 2011; Souza *et al.*, 2015). However, residual sexual activity should be expected in some apomictic genotypes (Reutemann *et al.*, 2022); sexual (Polygonum-type) embryo sacs have been observed, even at a high proportion (up to 0.93), in genotypes classified as apomictic (Worthington *et al.*, 2016).

The presence of A and B subgenomes in ‘humidicola-1’ accessions and allopolyploidy (AABBBB) is well supported (Vigna *et al.*, 2016; Worthington *et al.*, 2019; Tomaszewska *et al.*, 2021*b*). Worthington *et al.* (2019) proposed ‘humidicola-2’ accession 26146 to be autopolyploid (BBBBBB), while Vigna *et al.* (2016) proposed a composition similar to ‘humidicola-1’. We found evidence of a natural hybrid between these two groups (accession 16878), which suggests similar karyotypes in both subpopulations. Hexaploid and heptaploid accessions also appeared to be similarly frequent in both subpopulations (Tomaszewska *et al.*, 2021*b*).

### *Classification of the* Urochloa *hybrids*

In the centre of the agamic group, we identified the ‘agamic-admixed’ accessions (Fig. 4D). This cluster of accessions included hybrid accessions within the agamic group, and should not be confused with the ‘admixed’ accessions (Fig. 4C), which resulted from crosses between more distant *Urochloa* species. We provided our interpretation of the genetic make-up of these hybrids based on genetic markers in the results, but previous passport information was based on phenotypic characteristics alone. We think most of the ‘agamic-admixed’ accessions were wrongly classified as non-admixed in the passport data obtained from CIAT’s genebank databases, only cv. Cayman, BR02/1752, was classified as admixed (*Urochloa* spp.).

### *No subpopulations in* U. maxima

*U. maxima* is also known as *Panicum maximum* or *Megathyrsus maximus*. The genus *Urochloa* includes species previously classified under other taxonomic groups. We have opted to annotate all as *Urochloa*, as supported by recent work (Tomaszewska *et al.*, 2021*b*). Supporting this classification, we observed *U. maxima* was as genetically close to the *Urochloa* species in the agamic group as *U. humidicola*.

All *U. maxima* accessions (including the accession we sequenced as *U. humidicola* 26438, but which is in fact an unknown *U. maxima* accession) showed limited diversity (Fig. 4) and were assigned to a single subpopulation (‘maxima’) based on admixture and PCA. *U. maxima* was divided into two branches in the UPGMA tree, with three of the *U. maxima* accessions (6906, the unknown accession we received as 26438 and PI284156) separated from the rest. The accessions cover a great range of latitudes. The 12 accessions from CIAT were sourced from two distant regions (1100 km apart) in western Kenya and south interior Tanzania. The *U. maxima* accession PI284156, which was requested from the USDA collection later, originated from South Africa and showed a similar admixture to the others. Our results probably reflect no population structure in the species. Nine of the accessions analysed here were phenotypically characterized and separated into different clusters based on yield, protein and fibre composition, and nitrification rates (Villegas *et al.*, 2020).

### *Implications for* Urochloa *breeding*

Crosses between eight *U. brizantha*, one *U. decumbens* (cv. Basilisks) and one tetraploid *U. ruziziensis* (BRX 44-02) constitute the gene pool of the recurrent selection breeding programme at CIAT (Miles *et al.*, 2006). A similar breeding scheme is used at EMBRAPA (Barrios *et al.*, 2013), but we could not find information on the founders. The accession *U. decumbens* cv. Basilisks is used as single pollen donor in each cycle of recurrent selection in CIAT’s programme. The phylogenetic analysis in Triviño *et al.* (2017) placed the interspecific hybrids from the breeding programme (36061, 36087, BR02-, SX14-) between the polyploid *U. decumbens* and *U. brizantha.* This corresponds to the position of the natural *U. brizantha *× *U. decumbens* hybrids (‘agamic-admixed’) in our study. While the *U. brizantha* founders were selected based on phenotyping (Miles *et al.*, 2007), they are well distributed among the *U. brizantha* subpopulations. By placing our results into the UPGMA phylogenetic tree from Triviño *et al.* (2017), we can infer the subpopulations of the founders of CIAT’s programme: three were ‘brizantha/decumbens’ (16827, 16829 and 6297), three were ‘brizantha-2’ (16107, 16152 and 16296) and two were ‘brizantha-1’ (16126 and 6387). We also sequenced two founders of CIAT’s breeding programme (16152 and 16296), both resulting in ‘brizantha-2’, so verifying the approach. While our intention was to only include wild materials in the study, we accidentally sequenced BR02/1752 cv. Cayman, which is a product of the *U. brizantha *× *U. decumbens *× *U. ruziziensis* interspecific breeding programme and classified as a hybrid in the passport information. Approximately 75 % of cv. Cayman’s ancestry is from *U. ruziziensis* (light blue in Fig. 1) and ~25 % is *U. decumbens* ancestry (light purple in Fig. 3). While the markers of *U. ruziziensis* ancestry did not appear in other species, the markers of *U. ruziziensis* ancestry were also observed in brizantha-1 accessions. So, there may be some, but minor, *U. brizantha* (probably brizantha-1) ancestry in cv. Cayman. Most of the cv. Cayman genetic make-up is from a *U. ruziziensis* ancestor (Fig. 3).

The distance between ‘brizantha-1’ and the other *U. brizantha* groups may be useful to explore heterosis between distant *U. brizantha* crosses, and similarly between ‘humidicola-1’ and ‘humidicola-2’. Genetic maps generated for ‘humidicola-1’ and ‘humidicola-2’ suggest significant large structural variation between subpopulations (Worthington *et al.*, 2019). However, we found evidence of a natural hybrid between these two groups (accession 16878), which showed crosses between both subpopulations can be viable. The known ‘humidicola-2’ accessions are scarce, namely 26146 (Burundi), 26149 (Burundi), 6013 (South Africa), and 26570, 675, 679 and 682 (all unknown origin).

Incorporating genetic variation into breeding (elite × elite crosses), e.g. new resistance genes from ‘brizantha-1’, requires sorting through large combinations of alleles previously generated in wild × elite crosses. Even when candidate regions and alleles are clear, introgressions from the wild into elite germplasm may fail (McCouch *et al.*, 2020). Studies such as ours on plant genetic resources in genebanks help to clarify the genetic composition and relationships in the conserved materials, which can be used to develop approaches for addressing hybrid incompatibilities, reduced recombination or unexpected epistatic interactions.

## CONCLUSIONS

We established the population structure and evolution among and within the five *Urochloa* spp. that are used in the development of commercial forage cultivars using over 1 million markers, which allowed us to finely map differences between accessions. We identified ten subpopulations in total, which had no relationship to the geographical collection, and tentatively represented ten independent heterotic groups with distinctive adaptations (excluding the two admixed subpopulations) with application in breeding. Finding that some polyploid *U. decumbens* are more closely related to polyploid *U. brizantha* than to diploid *U. decumbens* supported an evolutionary model (previously proposed) where polyploidization established a tetraploid ancestor, from which polyploid *U. brizantha* and *U. decumbens* later diverged. In addition, we found two groups of apomictic polyploid *U. brizantha* accessions (brizantha-1 and -2) distant from each other and particularly from *U. decumbens.*

*Urochloa* diploids are often sexual, but natural sexual *Urochloa* polyploids are exceptionally uncommon (only the one *U. humidicola* accession in our study). Taking all these observations together, the subpopulation structure observed in the *Urochloa* sexual–apomictic multiploid complexes appears to be an archetypal case of geographical parthenogenesis (Hörandl and Hojsgaard, 2012; Hojsgaard and Hörandl, 2015), where polyploids exploit the advantages of apomixis, i.e. uniparental reproduction and clonal reproduction, to expand their geographical and ecological ranges, in a case very similar to *Paspalum* grasses (Ortiz *et al.*, 2013). One subpopulation, ‘humidicola-2’, had fewer accessions but included the only known sexual accessions in the species. We also observed one case of natural hybridization between both *U. humidicola* groups, suggesting a similar subgenome composition between subpopulations. Sexual accessions being exclusive to one of the subpopulations supports sexual reproduction being scarce and the importance of facultative apomixis in the evolution of *U. humidicola*.

## SUPPLEMENTARY DATA

Supplementary data are available online at https://academic.oup.com/aob and consist of the following: **Figure S1.** Cross-validation error and chosen value for number of groups for the complete dataset of 111 accessions, the subset of 67 accessions in the agamic group and the subset of 28 *U. humidicola* accessions. **Figure S2.** Admixture analysis for alternative values for number of groups in the complete set of 111 accessions. **Figure S3.** Admixture analysis for alternative values for number of groups in the subset of 67 accessions in the agamic group. **Figure S4.** Admixture analysis for alternative values for number of groups in the subset of 28 *U. humidicola* accessions. **Figure S5.** Summary diagram of the distribution of accessions in subpopulations according to the species and ploidy annotations. **Table S1.** Sample number, accession number, species, ploidy, subpopulation, architecture, collection location and PCA position for each of the 111 accessions used in this study.

mcac115_suppl_Supplementary_Figure_S1

mcac115_suppl_Supplementary_Figure_S2

mcac115_suppl_Supplementary_Figure_S3

mcac115_suppl_Supplementary_Figure_S4

mcac115_suppl_Supplementary_Figure_S5

mcac115_suppl_Supplementary_Table_S1

mcac115_suppl_Supplementary_Figure_Legend
