# Supplementary figures and images for: Diverged subpopulations in tropical Urochloa (Brachiaria) forage species indicate a role for facultative apomixis and varying ploidy in their population structure and evolution
Source: Ann Bot. 2022 Sep 16;130(5):657–69. doi: 10.1093/aob/mcac115 (PMC9670755; doi:10.1093/aob/mcac115)

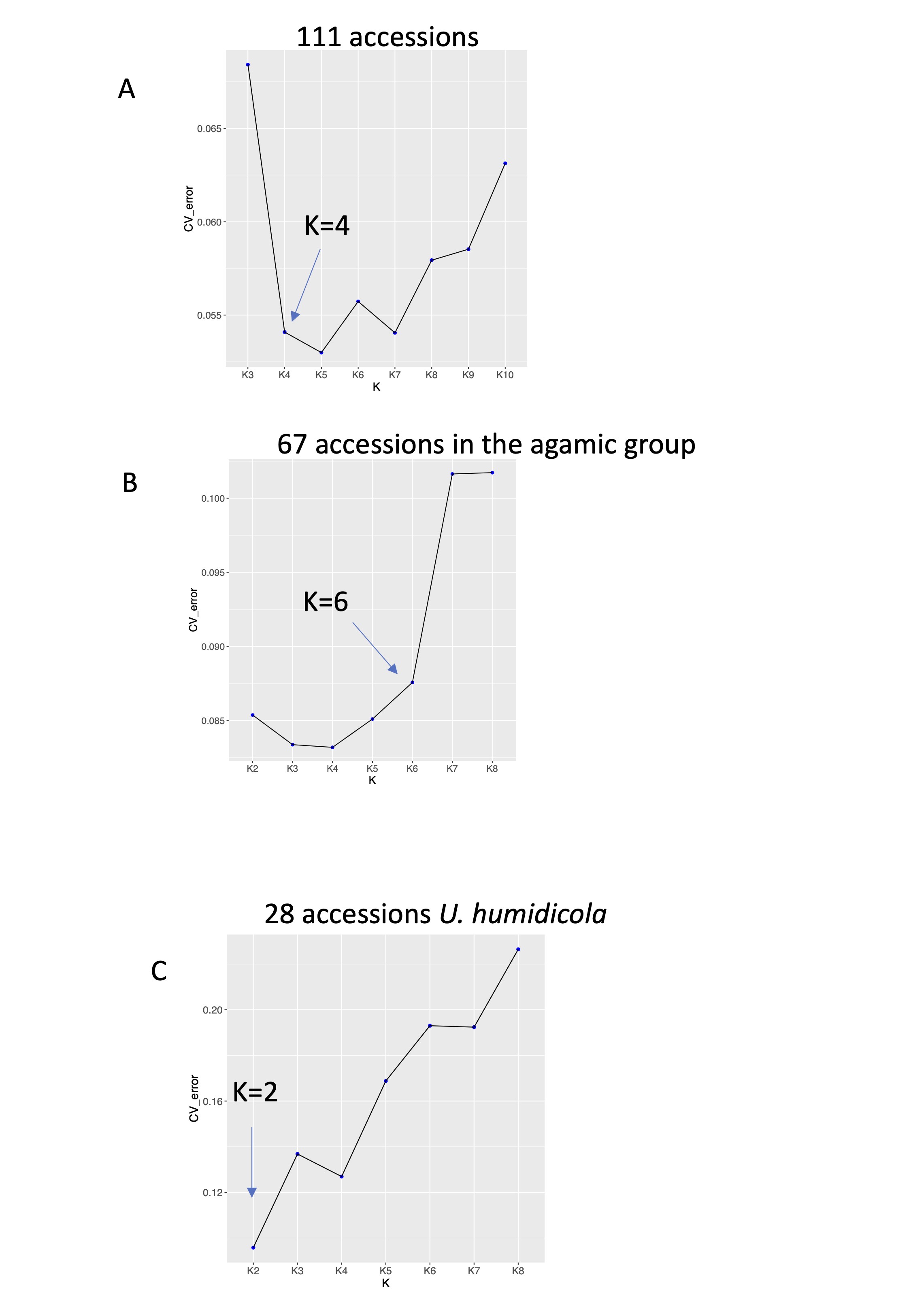

Supplement: mcac115_suppl_Supplementary_Figure_S1 [file mcac115_suppl_supplementary_figure_s1.jpeg]

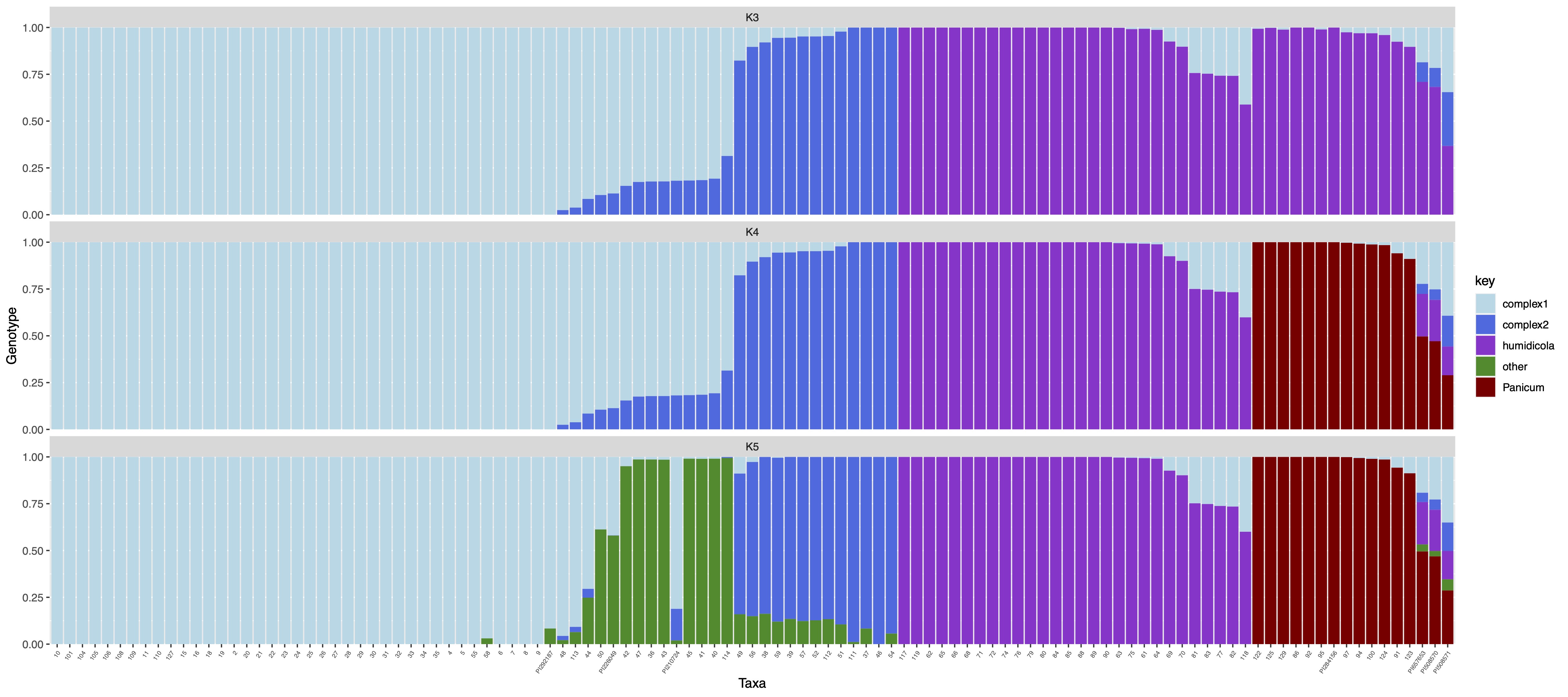

Supplement: mcac115_suppl_Supplementary_Figure_S2 [file mcac115_suppl_supplementary_figure_s2.jpeg]

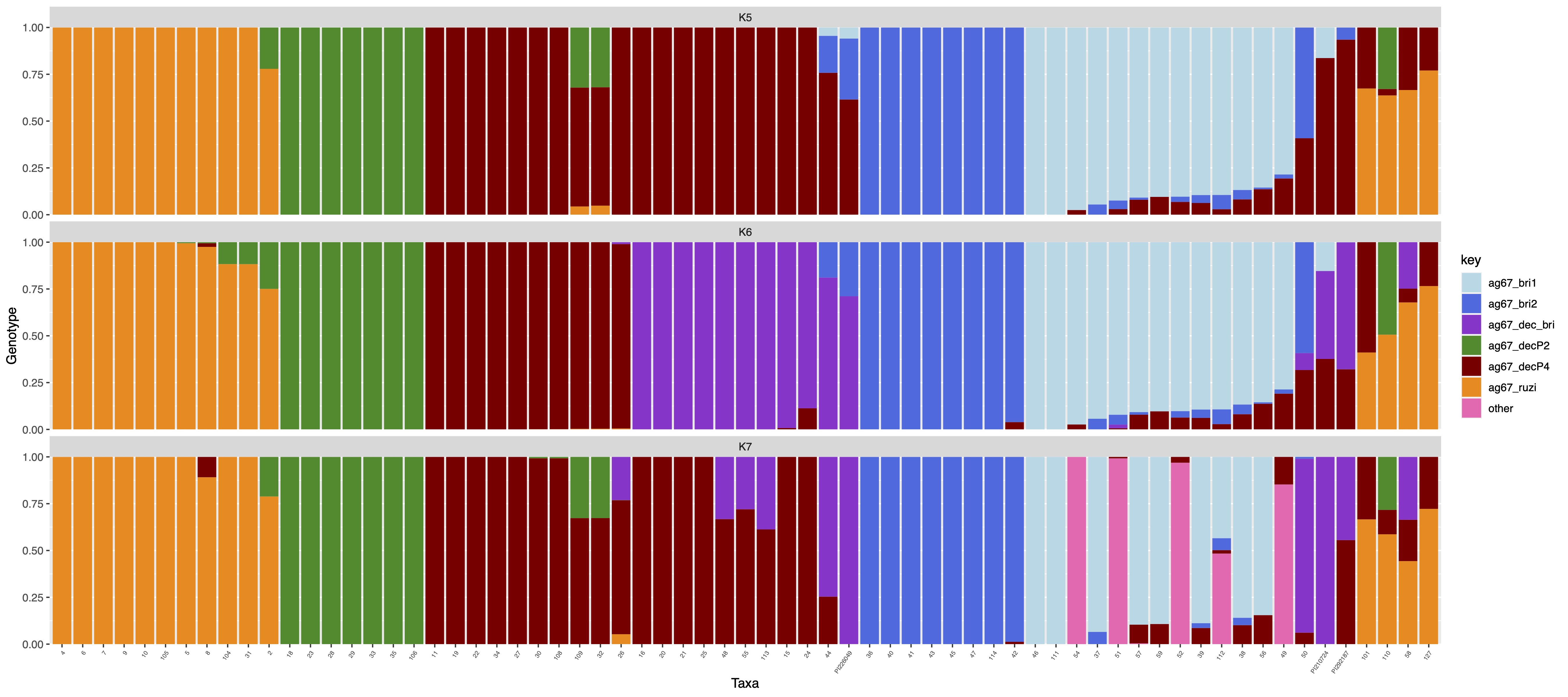

Supplement: mcac115_suppl_Supplementary_Figure_S3 [file mcac115_suppl_supplementary_figure_s3.jpeg]

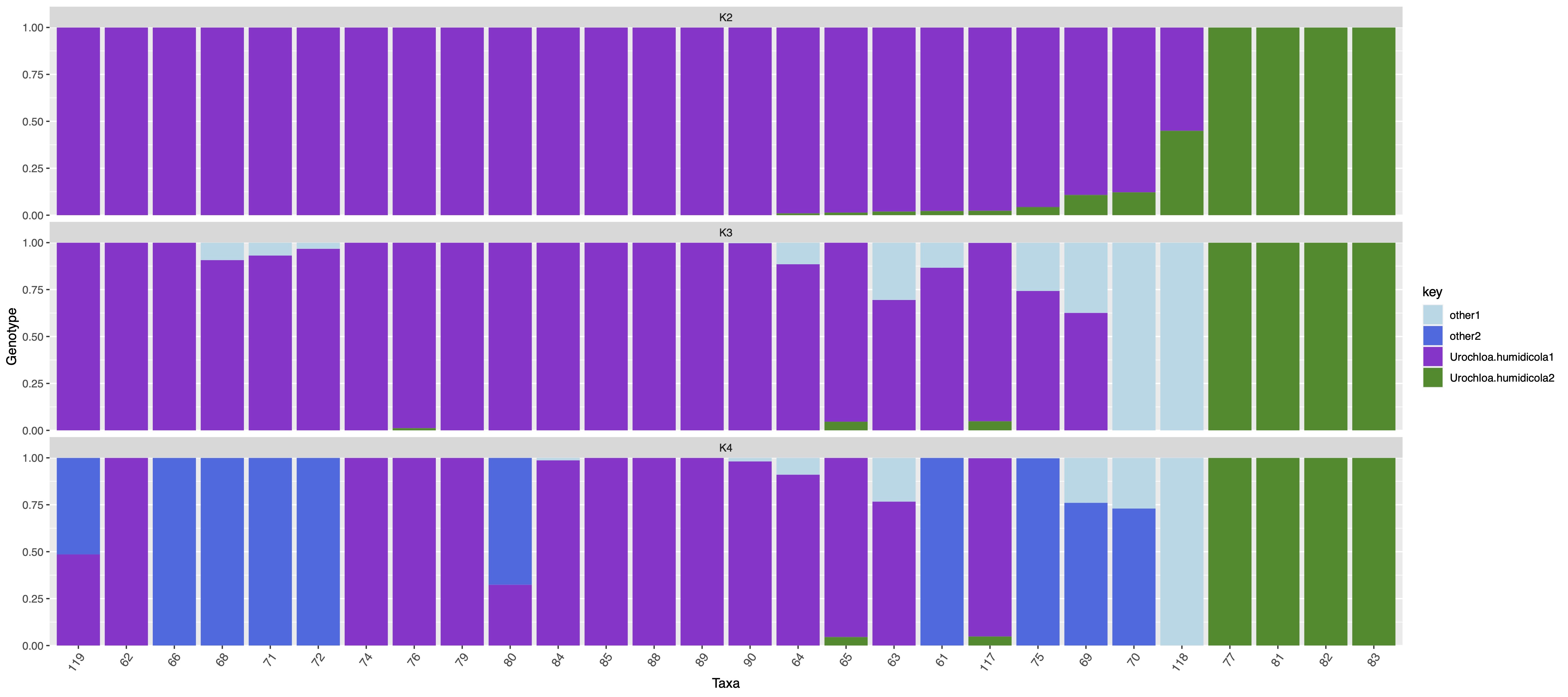

Supplement: mcac115_suppl_Supplementary_Figure_S4 [file mcac115_suppl_supplementary_figure_s4.jpeg]

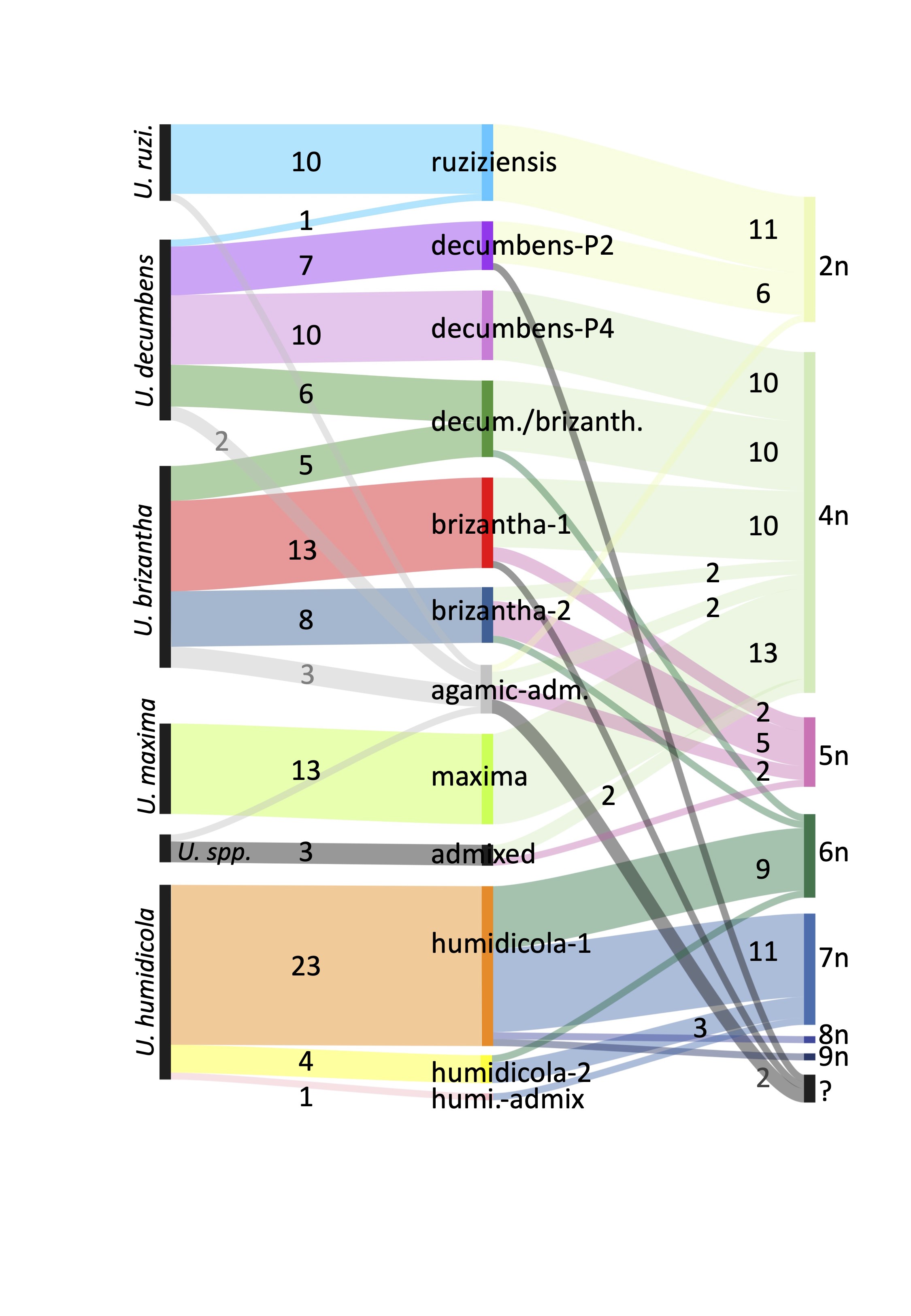

Supplement: mcac115_suppl_Supplementary_Figure_S5 [file mcac115_suppl_supplementary_figure_s5.jpeg]
