# Supplementary material for: Diverged subpopulations in tropical Urochloa (Brachiaria) forage species indicate a role for facultative apomixis and varying ploidy in their population structure and evolution
Source: Ann Bot. 2022 Sep 16;130(5):657–69. doi: 10.1093/aob/mcac115 (PMC9670755; doi:10.1093/aob/mcac115)
Supplement: mcac115_suppl_Supplementary_Figure_Legend [file mcac115_suppl_supplementary_figure_legend.docx]

Supplementary data captions

Figure S1: Cross-validation (CV) error and chosen value for number of groups (K) for the complete dataset of 111 accessions (A), the subset of 67 accessions in the agamic group (B), and the subset of 28 *U. humidicola* accessions (C). Cross-validation error is shown on the Y-axis (vertical) and the number of hypothetical populations on the X-axis (horizontal).

Figure S2: Admixture analysis for alternative values for number of groups (K = 3, 4 -selected-, and 5) in the complete set of 111 accessions. Numbered by “sample id”.

Figure S3: Admixture analysis for alternative values for number of groups (K = 5, 6 -selected-, and 7) in the subset of 67 accessions in the agamic group. Numbered by “sample id”.

Figure S4: Admixture analysis for alternative values for number of groups (K = 2 -selected, 3 and 4) in the subset of 28 *U. humidicola* accessions. Numbered by “sample id”.

Figure S5: Diagram summary of the distribution of accessions in subpopulations according to the species and ploidy annotations. The number on each stream represents the number of accessions in that division. Streams without a number represent a single accession.

Table S1. Sample number, accession number, species, ploidy, subpopulation, architecture, collection location, and PCA position for each of the 111 accessions used in this study.
